# Utilization of Pharmacological Ascorbate to Enhance Hydrogen Peroxide-Mediated Radiosensitivity in Cancer Therapy

**DOI:** 10.3390/ijms221910880

**Published:** 2021-10-08

**Authors:** Zain Mehdi, Michael S. Petronek, Jeffrey M. Stolwijk, Kranti A. Mapuskar, Amanda L. Kalen, Garry R. Buettner, Joseph J. Cullen, Douglas R. Spitz, John M. Buatti, Bryan G. Allen

**Affiliations:** Free Radical and Radiation Biology Program, Department of Radiation Oncology, The University of Iowa, Iowa City, IA 52242, USA; zain-mehdi@uiowa.edu (Z.M.); michael-petronek@uiowa.edu (M.S.P.); jeffrey-stolwijk@uiowa.edu (J.M.S.); krantiashok-mapuskar@uiowa.edu (K.A.M.); amanda-kalen@uiowa.edu (A.L.K.); garry-buettner@uiowa.edu (G.R.B.); joseph-cullen@uiowa.edu (J.J.C.); douglas-spitz@uiowa.edu (D.R.S.); john-buatti@uiowa.edu (J.M.B.)

**Keywords:** pharmacological ascorbate, radiation therapy, reactive oxygen species, hydrogen peroxide, radiosensitization

## Abstract

Interest in the use of pharmacological ascorbate as a treatment for cancer has increased considerably since it was introduced by Cameron and Pauling in the 1970s. Recently, pharmacological ascorbate has been used in preclinical and early-phase clinical trials as a selective radiation sensitizer in cancer. The results of these studies are promising. This review summarizes data on pharmacological ascorbate (1) as a safe and efficacious adjuvant to cancer therapy; (2) as a selective radiosensitizer of cancer via a mechanism involving hydrogen peroxide; and (3) as a radioprotector in normal tissues. Additionally, we present new data demonstrating the ability of pharmacological ascorbate to enhance radiation-induced DNA damage in glioblastoma cells, facilitating cancer cell death. We propose that pharmacological ascorbate may be a general radiosensitizer in cancer therapy and simultaneously a radioprotector of normal tissue.

## 1. Introduction

Of the approximately 1,800,000 new cases of cancer diagnosed in the United States each year, half will receive some form of ionizing radiation therapy (RT) as part of their therapy [1]. RT may be used alone or in combination with surgery and chemotherapy as treatment for various types of cancer. DNA damage induced by RT is central to its function in treating cancer. Ionizing radiation induces both DNA single- and double-strand breaks (SSB and DSB) that can lead to cell death due to increased genomic instability in cancer cells [2]. Additionally, RT can affect homeostasis and the redox environment of cells [3]. Since RT alone is often insufficient to provide tumor control for many cancer types, combinations with different chemotherapies have become ubiquitous, especially in the treatment of certain aggressive cancers [4,5]. Several cancer types that do not respond well to radiation and/or chemotherapy regimens have accelerated the development of additional radiation sensitizers.

In the 1970s, Cameron and Pauling introduced pharmacological ascorbate (P-AscH^−^) as a potential cancer therapy. P-AscH^−^ is IV-administered in gram doses (≈5 g to 100 g or more), yielding millimolar concentrations in blood. P-AscH^−^ is to be viewed as a drug; actually, it is a pro-drug for hydrogen peroxide [6]. Since the introduction of P-AscH^−^ into the cancer-arena, considerable research has been published highlighting its role not only as a cancer drug but also as a potential radiosensitizer. This research has produced promising results for some of the most aggressive cancer types including glioblastoma (GBM), non-small cell lung cancer (NSCLC), and pancreatic cancer [4,5,7]. Historically, these aggressive cancers respond poorly to radiation and chemoradiation treatment approaches, accentuating the need to explore alternative and adjuvant therapies like P-AscH^−^.

GBM is the most common primary brain cancer in the U.S.A. accounting for 70% of the 22,500 new malignant primary brain tumors diagnosed each year [8]. The current treatment regimen of surgical resection, RT, and chemotherapy with temozolomide (TMZ) is exceedingly aggressive, with up to 54% of patients experiencing seizures 5 days postoperatively. The median survival remains less than two years [9,10,11,12].

A more common and difficult-to-treat cancer type is lung cancer. It is the leading cause of cancer-related death in the U.S.A., with approximately 200,000 new cases and nearly 200,000 deaths each year [13]. Non-small cell lung cancer (NSCLC) accounts for 80% of lung cancer cases. It is treated in a variety of ways including RT, chemotherapy, targeted therapy, immunotherapy, and combination treatments. Despite treatment algorithms, the five-year survival for NSCLC remains only 15% [14].

Additionally, pancreatic cancer is the fourth deadliest cancer, with more than 48,000 deaths in the U.S.A. [15]. Five-year survival is only 10.8%. A major consideration in pancreatic cancer is local disease, as 30% of patients succumb to local disease progression. Thus, improving current RT regimens to combat local disease is an important avenue in treating these patients [16].

These cancers are examples of some of the most difficult to treat, with very poor five-year prognoses. However, there are encouraging data on the potential of P-AscH^−^ as an adjuvant to radiation and chemotherapy therapy. This review presents the current state of knowledge on P-AscH^−^ as an adjuvant to standard-of-care therapies, where it acts as a radiosensitizer via H_2_O_2_-mediated cytotoxicity in cancer cells and tissue and as a radioprotector of normal cells and tissues. 

## 2. History of the Use of P-AscH^−^ as a Cancer Drug

P-AscH^−^ was first assessed as a potential cancer treatment in the 1970s by Cameron and Pauling, who hypothesized that P-AscH^−^ improved host resistance to metastatic cancer growth [7,17]. At the time, the theory supporting the use of ascorbate was that terminal cancer patients presented with scurvy-like symptoms due to low ascorbate levels. Therefore, the administration of ascorbate could lead to potential benefits [7]. The two researchers conducted clinical trials comparing overall survival between advanced cancer patients treated solely with P-AscH^−^ and untreated matched historical controls [18,19]. These trials demonstrated the safety of intravenous (IV) P-AscH^−^ and its potential efficacy as a cancer therapy. Specifically, 100 terminal cancer patients were given 10 g of IV P-AscH^−^ for 10 days, followed by 10 g of oral supplementation and were matched with 1000 historical control patients. While there was some variability depending on the cancer type, the data showed a four-fold increase in the survival of patients treated with P-AscH^−^ compared to matched controls [20,21].

In a randomized, double-blinded clinical trial involving 123 advanced cancer patients with tumors in various primary sites, oral ascorbate showed no therapeutic advantage when compared to placebo (60 patients receiving a placebo, and 63 administered oral P-AscH^−^, 10 g) [22]. This study was repeated in colorectal carcinoma patients with similar negative results [23]. Because these trials failed to support Cameron and Pauling’s original results, interest in ascorbate as a potential treatment option for cancer patients waned.

Lack of interest in P-AscH^−^ as a cancer therapy continued until the late 1990s and early 2000s. During this time, comparative pharmacokinetic analysis between oral and IV administration of P-AscH^−^ uncovered that significantly greater concentrations of AscH^−^ in blood could be achieved via IV administration, safely reaching concentrations of 25–30 mM, compared to the oral route that allows a maximal concentration of approximately 220 μM [24,25,26,27]. With this discovery, interest in high-dose, IV-administered P-AscH^−^ reemerged as a potential therapeutic avenue for cancer treatment. 

Since then, various preclinical and clinical trials have explored the therapeutic benefit and mechanism of P-AscH^−^ as a cancer treatment, either alone or in combination with standard-of-care radiation and/or chemotherapy treatments. Through explorative mechanistic studies, hypotheses were developed that P-AscH^−^ functions in cancer therapy via reactive oxygen species (ROS) and oxidative damage to cancer cells. While various contentions were made to this potential mechanism, only recently has significant progress been made demonstrating the importance of oxidative damage via P-AscH^−^ administration. Nonetheless, clinical trials prior to this exploration demonstrated the efficacy of this therapy. 

In 2004, a small study in seven patients with renal cell carcinoma, pancreatic cancer, colorectal cancer, non-Hodgkin’s lymphoma, or breast cancer demonstrated the safety of P-AscH^−^ [28]. In the two renal cell carcinoma case-study patients, P-AscH^−^ alone resulted in the resolution of disease. In a late-stage colorectal carcinoma patient, a combination of chemotherapy (5-FU and leucovorin) followed by P-AscH^−^ (plasma ascorbate concentration of 20 mM) resulted in resolution of the disease with minimal side effects associated with P-AscH^−^. In a pancreatic cancer patient that was refractory to gemcitabine, treatment with P-AscH^−^ combined with gemcitabine decreased CA-19-9 and slowed tumor growth. In a non-Hodgkin’s lymphoma patient who refused chemotherapy or radiation, P-AscH^−^ alone (15 g infusions) resulted in complete resolution of the disease. Another non-Hodgkin’s lymphoma patient who discontinued chemotherapy because of associated side effects was administered P-AscH^−^ and showed no evidence of disease within three months of treatment, with complete remission by the end of 11 months of treatment. In addition, an end-stage, metastatic breast carcinoma patient treated with daily P-AscH^−^ (maximum dosage of 100 g/d) reported a significant reduction in bone metastases and a marked improvement in quality of life within three months from the start of treatment. The encouraging results described in these selected case reports generated renewed investigation of P-AscH^−^ for use in cancer treatment.

In the 2010s, early-phase clinical trials showed potential efficacy of combining P-AscH^−^ with chemotherapy and radiation. Phase I trials in metastatic pancreas cancer combining P-AscH^−^ with erlotinib and gemcitabine or gemcitabine alone found the combinations to be safe, with toxicity similar to chemotherapy alone and potential promising clinical outcomes [29,30].

Furthermore, in an important development for pancreatic cancer therapy, we worked with colleagues at our institution to conduct a phase I clinical trial involving 14 locally advanced pancreatic cancer patients treated with gemcitabine, radiation, and P-AscH^−^, who demonstrated an overall survival (OS) of 21.7 months (an increase from an institutional average of 12.7 months) and progression-free survival (PFS) of 13.7 months (an increase from 4.6 months) [15]. In addition, three patients were disease-free 5 years after treatment. Beyond a potential therapeutic gain, this study also provided evidence that P-AscH^−^ decreased radiation-induced bowel injury in pre-clinical animal models.

In addition to pancreatic cancer, a phase I clinical trial of 11 glioblastoma patients who were treated with standard-of-care treatments (temozolomide and RT) plus P-AscH^−^ showed a higher OS of 18 months and a higher PFS of 9.4 months compared to historical medians of 14.6 months and 7 months, respectively [31]. Moreover, these data also included eight GBM patients with an unmethylated O-6-methylguanine-DNA methyltransferase (MGMT) promoter, which generally have worse prognoses, with a historic median OS of 12 months. The median OS in the trial for these patients was 23 months, and the median PFS was 10 months [31,32]. Although the investigation of efficacy is not a goal of phase I trials, these observations are very encouraging.

Due to the tumor- and therapy-specific variability in the apparent efficacy of P-AscH^−^, there is a need for continued preclinical and clinical research exploring the underlying mechanism of P-AscH^−^-mediated cytotoxicity. Nonetheless, these clinical trials are promising for the use of P-AscH^−^ as an adjuvant to the standard of care. Exciting is that these early results support the concept that P-AscH^−^ may be useful as a radiosensitizer and that it may simultaneously serve as a radioprotector of vulnerable normal tissue.

## 3. Radiation-Induced Injury in Cancer and the Role of Hydrogen Peroxide

The role of P-AscH^−^ as a radiosensitizer and its proposed mechanism of H_2_O_2_-mediated cytotoxicity are based on the generation of reactive oxygen species (ROS) following RT. RT exerts its effects on DNA either by directly damaging DNA or by indirectly interacting with water molecules to form oxidizing free radicals that in turn lead to DNA damage [33]. Because low linear energy transfer (LET) radiation (i.e., photons) is the primary clinical RT modality and because water makes up 50–60% of the adult body weight, RT’s indirect mechanism of DNA damage dominates, wherein ionization of water generates oxidizing chemical species (i.e., ROS) that will damage target biomolecules, namely, proteins, lipids, and most significantly, DNA [34,35].

ROS can be divided into two broad groups: free radicals and non-radicals. Some free radicals are highly reactive species, including superoxide (O_2_^•−^), hydroxyl radical (HO^•^), nitric oxide (NO^•^), peroxyl radicals (ROO^•^), alkoxyl radicals (RO^•^), etc., whereas, non-radical ROS species include hydrogen peroxide (H_2_O_2_), singlet oxygen (^1^O_2_), organic hydroperoxides (ROOH), peroxynitrite (ONOO^−^), etc. [36]. Increased levels of ROS have been detected in almost all types of cancers and may play a critical role in mediating tumor development and progression [37,38,39]. Elevated ROS levels in cancer can be attributed to mitochondrial dysfunction, increased activity of NAPDH oxidases, increased expression of oncogenes, and overwhelmed or downregulated metabolic pathways that detoxify ROS [36,38,40,41,42,43,44]. The type, location, and concentration of the free radicals generated following RT can activate specific downstream pathways that can affect cellular functions in cancer cells [36].

ROS generated by RT are indiscriminate of cancerous and non-cancerous tissues. Thus, RT-induced ROS can accelerate tumor cell killing but also cause normal tissue injury via impairment of mitochondrial dysfunction and genomic instability [45]. There are several cancers treated with RT where RT-induced, dose-limiting complications occur in normal tissue that limit the therapeutic benefit of RT alone. Specifically, the lungs are one of the most sensitive tissues to RT; therefore, their increased susceptibility to RT damage limits treatment. Studies in lung and esophageal cancers have shown that RT effects on the lung can be seen as acute toxicity (hours to days following RT) and late injury (months to years following RT) including pulmonary fibrosis, necrosis, and atrophy [46]. During standard RT treatment for brain cancers such as fractionated partial- and whole-brain radiation treatment (PBRT and WBRT), healthy brain tissue is inevitably exposed, resulting in side effects, such as learning and cognitive deficits, including memory impairment, neurological deficits, increased intracranial pressure, and progressive dementia [47,48,49,50]. Therefore, the indiscriminate nature of RT-induced ROS and cellular damage underscores the necessity for developing therapies, such as P-AscH^−^, to selectively enhance the effects of radiation on cancer cells, while simultaneously protecting normal tissues from RT-induced toxicity [51].

## 4. P-AscH^−^ and the Flux of Hydrogen Peroxide

While RT-induced ROS formation is a fundamental physiochemical mechanism in cancer cell killing, ROS generation is also central to the selective toxicity of P-AscH^−^. As evidenced by early pharmacokinetic work, the anti-cancer effects of P-AscH^−^ occur primarily at supraphysiological plasma concentrations [24]. Humans have evolved to tightly control the concentration of ascorbate following oral ingestion through the processes of intestinal absorption, ascorbate transport, and renal excretion [24,27]. Consuming ≥ 200 mg of ascorbate decreases its intestinal absorption [52]. At a plasma concentration of ≈60 mM, the ascorbate transporter, sodium vitamin C transporter 2 (SVCT2), is saturated and approaches its *V*_max_, and renal absorption declines, with excess ascorbate being excreted in the urine [24,27,53]. Alternatively, intravenous administration of P-AscH^−^ bypasses these control mechanisms resulting in supraphysiologic plasma and tissue ascorbate concentrations (mM) that selectively induce cancer cell death [54]. Intracellularly, concentrations of H_2_O_2_ between 0.001 and 10 μM are involved in signal transduction pathways required for cell survival [54]. At these low intracellular concentrations, H_2_O_2_ can cause reversible oxidation of redox-sensitive transcription factors and enzymes [54]. However, P-AscH^−^ has been shown to generate fluxes of H_2_O_2_ beyond the detoxification capacity of the cell, leading to cell death [55,56]. 

The proposed mechanism of P-AscH^−^-mediated cancer cell toxicity is via the redox-active metal-mediated formation of H_2_O_2_ (**Rxn 1**) [5,6,57].

AscH^−^ + O_2_ + H^+^_aq_ → H_2_O_2_ + DHA (1)

where DHA is dehydroascorbic acid, the two-electron oxidation product of AscH^−^. Rxn 1 is the overall reaction with many steps in the mechanism, vide infra.

Pioneering work by Chen et al. in 2005 identified P-AscH^−^ as a pro-drug for the generation of H_2_O_2_ in tissues [58]. In the presence of redox-active metals, ascorbate is oxidized forming the ascorbate radical (Asc^●−^) and a reduced metal [58,59]. The reduced metal then transfers an electron to molecular oxygen, forming superoxide (O_2_^●−^). Superoxide can be converted into H_2_O_2_ via superoxide dismutases. H_2_O_2_ formation is linearly related to Asc^●−^ formation, with detectable H_2_O_2_ formation occurring when [Asc^●−^] exceeds 100 nM [6]. Because H_2_O_2_ is uncharged, it can passively diffuse across membranes; however, certain aquaporins, termed peroxiporins, actively import H_2_O_2_ into cells; they are the primary facilitators of cellular uptake of extracellular H_2_O_2_. This gating of import modulates the intracellular effects of extracellular H_2_O_2_, specifically, intracellular oxidative distress that can lead to cell death [54,60]. This hypothesized H_2_O_2_-dependent mechanism for the selectivity of P-AscH^−^ was confirmed with intracellular as well as extracellular catalase, an enzyme that removes H_2_O_2_ converting it to water and oxygen. The inclusion of catalase prior to treatment with P-AscH^−^ has been shown to mitigate P-AscH^−^ toxicity [6]. Conversely, the introduction of superoxide dismutase prior to treatment with P-AscH^−^ enhanced intracellular H_2_O_2_ formation by P-AscH^−^ [6]. Thus, ROS generation is critical for the toxicity of P-AscH^−^.

With ROS generation being crucial for the mechanisms of action for both RT and P-AscH^−^, several preclinical studies have explored the potential radio-sensitizing role of P-AscH^−^. In vitro studies using NSCLC and GBM cells demonstrated that treatment with 40 pmol cell^−1^ (2 mM) of P-AscH^−^ for 1 h in combination with chemoradiation (5 μM of carboplatin or TMZ) for 1 h and 2 gray (Gy) ionizing radiation (IR) led to significant reduction in clonogenic cell survival compared to P-AscH^−^ or chemoradiation alone [5]. Conversely, no significant difference was observed in normal human bronchial epithelial cells (HBEpC) and normal human astrocytes (NHA) treated with P-AscH^−^ and chemoradiation as compared to the chemoradiation group, suggesting that the combined treatment of P-AscH^−^ and chemoradiation was selectively toxic to cancer cells [5]. To support the notion that P-AscH^−^ exhibits a functional radiosensitizer role, treatment of pancreatic cancer cells with P-AscH^−^ showed that administration either an hour before or an hour after IR was more cytotoxic than when P-AscH^−^ was administered six hours after IR. These data suggest that the synergistic effects of P-AscH^−^ and IR may be due to enhanced propagation of ROS following IR [4]. Similar experiments have shown that P-AscH^−^ is selectively toxic to cancer cells and enhances radiation sensitivity in non-small cell lung cancer, glioblastoma, sarcoma, and pancreatic cancer, while remaining relatively innocuous to normal cells [4,5,55,61].

**Figure 1:**
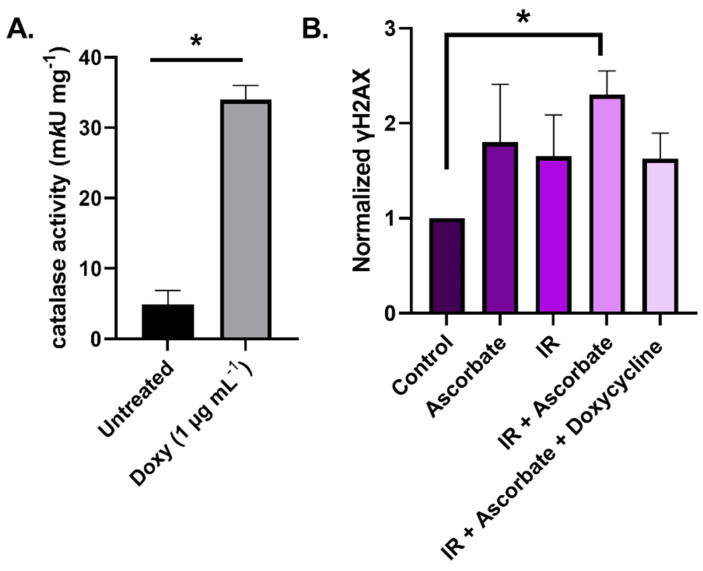
P-AscH^−^ enhances DNA damage induced by ionizing radiation (IR) in GBM cells, as seen by activation of γH2AX. (**A**) Catalase activity is increased by ≈8-fold in catalase-overexpressing U87 cells. * *p* < 0.05. Doxy (doxycycline) initiates the cellular overexpression of catalase. (**B**) IR + ascorbate increases DNA damage, as seen by normalized γH2AX expression in U87 cells. Ascorbate concentrations were 10 pmol/cell (2 mM). The dose of IR was 4 Gy. Doxycycline treatment was 1 μg/mL. Unpaired Student’s *t*-test was used to compare IR + P-AscH^−^ with control, * *p* < 0.05.

These data, vide supra, indicate that P-AscH^−^-induced fluxes of H_2_O_2_ are central to its ability to function as a radiosensitizer. Thus, we hypothesized that P-AscH^−^ would enhance IR-induced DNA damage. We tested if P-AscH^−^ can enhance IR-induced DNA damage via increase flux of H_2_O_2_ using doxycycline-inducible catalase-overexpressing U87 GBM cells (See Materials and Methods section for protocol details.). Catalase activity was eight times that of controls in the overexpressing cells, as shown in Figure 1A. Cells treated with P-AscH^−^ and IR individually revealed an increase in double-stranded DNA damage, as assessed by phosphorylated H2AX (γH2AX), relative to sham-treated cells, as shown in Figure 1B. The combination of P-AscH^−^ and IR resulted in significantly more γH2AX than in untreated cells, as shown in Figure 1B. To investigate the role of H_2_O_2_, doxycycline inducible catalase-overexpressing U87 cells were treated with P-AscH^−^, RT, and doxycycline; we observed no difference in γH2AX compared to the amount measured after treatment with IR alone, indicating that H_2_O_2_ may be critical for P-AscH^−^ to function as a radiosensitizer. These results warrant further investigation into the ability of P-AscH^−^ to enhance IR-induced DNA damage with regards to both time and dose dependence. 

One hypothesis to explain how high doses of ascorbate selectively kill cancer cells while having minimal effect on non-malignant cells proposes that cancer cells have increased uptake of dehydroascorbic acid (DHA), the two-electron oxidation product of ascorbate, via increased expression of GLUT1 transporters [62]. Increases in DHA in cancer cells could deplete cellular glutathione levels and thereby augment cancer cell oxidative distress [62]. However, later studies showed that competitive inhibition of GLUT transporters with 2-deoxy-D-glucose did not suppress P-AscH^−^ toxicity but rather enhanced P-AscH^−^ toxicity [5]. Thus, these data along with the kinetic arguments presented by Doskey et al. [56] rule out this mechanism as a significant contributor to the toxicity of P-AscH^−^. 

**Figure 2:**
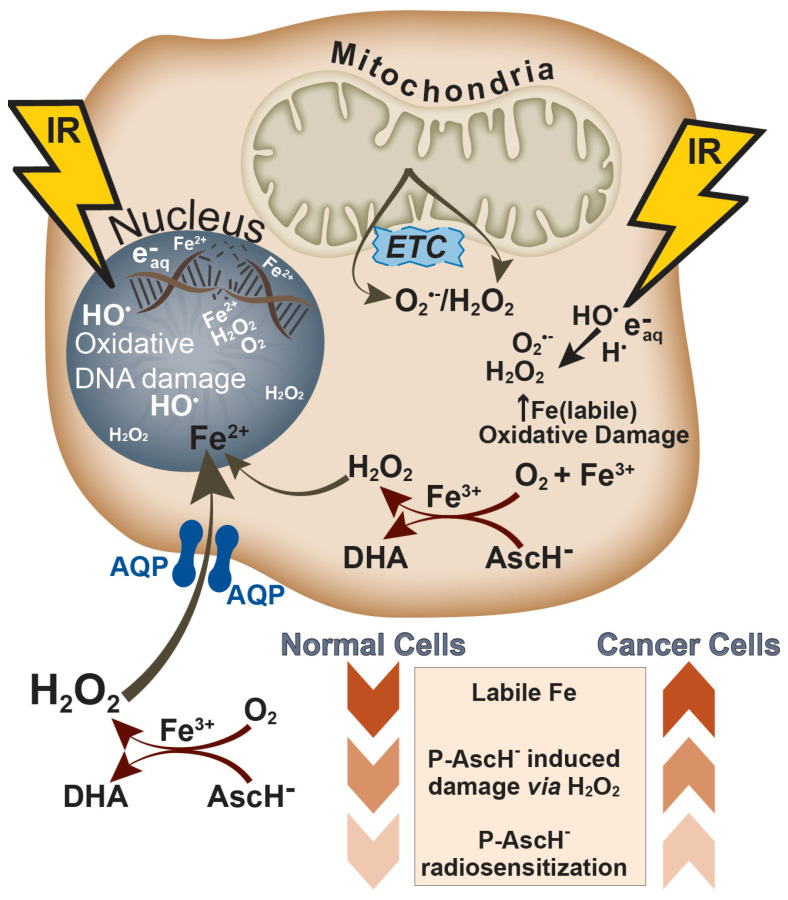
Proposed mechanism of action of P-AscH^−^. With redox-active, labile iron as a catalyst, P-AscH^−^ generates high fluxes of H_2_O_2_, intracellularly and, especially, extracellularly. Extracellular H_2_O_2_ is readily brought into cells via aquaporins (AQP); some AQPs are referred to as peroxiporins as they efficiently take up, i.e., gate, H_2_O_2_. This pool of redox-active, labile iron also reacts with H_2_O_2_ producing the extremely oxidizing HO^∙^. Because this pool of iron is mostly loosely coordinated to biomolecules, such as DNA, site-specific oxidative damage occurs. IR brings about complementary oxidative damage, via production of reactive species from the radiolysis of water, as well as direct damage to DNA, both double-strand and single-strand breaks. The DNA damage produced by P-AscH^−^ via H_2_O_2_ and redox-active, labile iron synergizes with intracellular Fe^2+^/Fe^3+^ to facilitate the propagation of oxidative events, leading to enhanced RT-induced DNA damage. IR, O_2_^∙−^, and H_2_O_2_ are each able to increase the level of labile iron, providing a feed-forward set of events. The ambient levels of labile iron and the steady-state levels of H_2_O_2_ and O_2_^∙−^ are higher in cancer cells than in normal cells, leading to greater radiosensitization in the former than in normal cells. In fact, P-AscH^−^ appears to serve as a radio-protector to normal cells and tissues.

Our hypothesis for the selective toxicity of P-AscH^−^ to cancer cells and its relative harmlessness to non-malignant cells is centered on the altered redox metabolism of cancer cells. We have proposed that fundamental defects in cancer cell mitochondrial metabolism result in increased steady-state levels of reactive oxygen species, including O_2_^●−^ and H_2_O_2_ (Figure 2) [5]. We further propose that redox-active, labile metals, specifically iron (Fe), are central to the toxicity of P-AscH^−^ [39,43,57]. This iron serves as a multiplier of oxidative damage from ROS by: (1) facilitating an increased flux of H_2_O_2_ by catalyzing the oxidation of ascorbate; and (2) “activating” this H_2_O_2_, via the Fenton reaction (vide infra), to form HO^•^ that in turn oxidizes important biomolecules, such as DNA. 

Below we present some of the chemical details showing how P-AscH^−^ leads to production of H_2_O_2_ and its interplay with intracellular Fe. 

## 5. Targeting Intracellular Iron to Enhance Radiation-induced Oxidative Damage

### 5.1. Disruption of Intracellular Iron Metabolism by P-AscH^−^

Cancer cells often accumulate Fe to levels above that of corresponding normal cells via increased iron import and decreased export [63,64,65]. This increased Fe content in tumors can turn P-AscH^−^ into a pro-oxidant [59]. Chemically, ascorbate (AscH^−^) is an outstanding one-electron reductant. In appropriate coordination environments, AscH^−^ can provide an electron to reduce Fe^3+^ to Fe^2+^ (**Rxn 2**) [59]:

AscH^−^ + Fe^3+^ → Asc^●−^ + Fe^2+^ (2)

However, the enhanced flux of H_2_O_2_ produced by P-AscH^−^ is also able to affect the distribution of intracellular iron [5]. H_2_O_2_ can interact with catalytically active Fe^2+^ via classic Fenton chemistry to generate hydroxyl radicals and further enhance detrimental oxidations (**Rxn 3**) [66]:

Fe^2+^ + H_2_O_2_ → Fe^3+^ + HO^●^ + OH^−^ (Fenton reaction) (3)

Fe^2+^ in near-neutral pH environments will react directly with oxygen, producing oxidants oxidants as well as superoxide (**Rxn 4**) [67]:

Fe^2+^ + O_2_ → [Fe^2+^-O_2_ ←→ Fe^3+^-O_2_^●−^] → Fe^3+^ + O_2_^●−^ (4)

The loosely bound, redox-active Fe^3+^, often referred to as labile iron, can in principle be re-reduced by superoxide (**Rxn 5**) [67]:

Fe^3+^ + O_2_^●−^ → Fe^2+^ + O_2_ (5)

However, Rxn 5 is an exceptionally rare event, with Rxn 2, as well as other cellular reduction processes, dominating. The more important reaction of superoxide, via its protonated form HO_2_^•^, is the oxidation of enzymes containing [4Fe-4S] clusters that have a solvent-accessible iron, such as dehydratases. This results in the release of Fe^2+^ into the redox active, labile iron pool (**Rxn 6**) [68].

HO_2_^●^ + [4Fe-4S]^2+^ + H^+^_aq_ → Fe^2+^ + H_2_O_2_ + [3Fe-4S]^1+^ (6)

The released Fe^2+^ facilitates oxidative damage to cells, including DNA damage [69,70,71]. 

In addition to interacting with catalytically active Fe^2+^ via Rxns 2 and 3, H_2_O_2_ generated by P-AscH^−^ can oxidize these [4Fe-4S]-containing proteins, e.g., aconitase (**Rxn 7**) [5,72,73].

H_2_O_2_ + [4Fe-4S]^2+^ → Fe^2+^ + HO^●^ + OH^−^ + [3Fe-4S]^1+^ (7)

Similar to what is observed for superoxide, the H_2_O_2_-dependent oxidation of [4Fe-4S]^2+^ is likely due to a chemical interaction with the solvent-exposed Fe^2+^ coordination site, leading to the release of a freely-chelatable Fe^2+^ [74,75]. The rate constant for Rxn 7 is on the order of 1000 times smaller than Rxn 6 at neutral pH; however, the intracellular steady-state level of H_2_O_2_ is much greater than that of O_2_^•−^/HO_2_^•^ [76,77].

Aconitase is a TCA cycle intermediate that contains a [4Fe-4S]^2+^ cluster that is central to its enzymatic activity [74,75]. It has been previously shown in GBM and NSCLC cells that P-AscH^−^ can blunt aconitase activity [5]. This effect was ameliorated by catalase overexpression, consistent with the generation of H_2_O_2_ by P-AscH^−^ as a species that contributes significantly to the inactivation of aconitase. 

The Fe^2+^ ion released can then be oxidized by O_2_ (Rxn 4), which will contribute to the propagation of oxidation reactions [67]. Taken together, the interplay of H_2_O_2_ and O_2_^•−^ with catalytically active, intracellular iron aids in the further propagation of oxidative damage initiated by P-AscH^−^. These disruptions of Fe metabolism are proposed to be central to the selective radiosensitization of cancer cells by P-AscH^−^.

### 5.2. Interactions of Iron and Ionizing Radiation

Redox-active Fe^2+/3+^ can also be affected by IR; an increase in catalytically active Fe^2+/3+^ stimulated by P-AscH^−^ would further facilitate intracellular Fe radiochemistry. Fe^2+^ can be readily oxidized by the reactive chemical species produced by IR, as first described by Fricke and Morse in 1927 [78]. This reaction has since been adopted as a means to determine radiation dose in aqueous solutions. However, it may be applicable to cellular systems as well. 

Immediately following dose deposition using low LET IR (e.g., photons), the radiolysis of H_2_O occurs, leading to the ionization of water (**Rxn 8**) [33]:

H_2_O + IR → H_2_O^●+^ + e_aq_^−^ (8)

The aqueous electron can reduce Fe^3+^ and has been shown to lead to the reduction and release of Fe from ferritin, which results in an open Fe-binding site within the protein (**Rxn 9**):

e_aq_^−^ + Fe^3+^ → Fe^2+^ (9)

This reaction has been elucidated via pulse radiolysis but has not yet been fully examined in vitro. 

The H_2_O^●+^ of Rxn 8 will immediately deprotonate to yield the very oxidizing hydroxyl radical (**Rxn 10**):

H_2_O^●+^ → HO^●^ + H^+^_aq_ (10)

The aqueous electron of Rxn 8 will very rapidly react with O_2_, leading to the generation of superoxide (**Rxn 11**):

e_aq_^−^ + O_2_ → O_2_^●−^ (11)

Ionizing radiation can also simply result in homolytic bond cleavage (**Rxn 12**):

H_2_O + IR → HO^●^ + H^●^ (12)

Following the initial physiochemical steps associated with the hydrolysis of H_2_O, Fe^2+^ can be readily oxidized by several chemical species. These chemical steps are typically used to estimate the radiation dose to water (D_w_) and are referred to as Fricke dosimetry [78,79]. 

The hydrogen atom (H^•^) will also react with O_2_ to form a hydroperoxyl radical, i.e., protonated superoxide (**Rxn 13**), which can oxidize Fe^2+^ (**Rxn 14**):

H^+^_aq_ + O_2_ → HO_2_^●^ (13)

HO_2_^●^ + Fe^2+^ + H^+^_aq_ → Fe^3+^ + H_2_O_2_ (14)

H_2_O_2_ is now available for the Fenton reaction, Rxn 3, to produce a hydroxyl radical. Lastly, each hydroxyl radical produced either via the Fenton reaction (Rxn 3) or by the radiolysis of H_2_O can oxidize a Fe^2+^ (**Rxn 15**):

HO^●^ + Fe^2+^ + → Fe^3+^ + OH^−^ (15)

Before considering the cellular effects of this chemical reaction, it is important to note the timescale on which these reactions occur [80]. There is a bi-phasic oxidation of Fe^2+^ in solution, with an initial burst of oxidation occurring in ≈10^−8^ s and maximizing at ≈10^−6^ s. The first burst of Fe oxidation is the result of HO_2_^•^-mediated oxidation (Rxn 14). There is a second burst of oxidation that begins at ≈10^−4^ s and reaches a maximum at ≈10^−2^ s. The second burst of Fe oxidation is the result of H_2_O_2_- and HO^•^-mediated events; this HO^•^ arises from the Fenton reaction, Rxn 3. Because these events occur so rapidly, Fricke dosimetry is a very useful tool for estimating the radiation dose. This chemistry illustrates the rich interplay between ROS produced by the radiolysis of water and iron, which will lead to the oxidation of cellular biomolecules (Figure 2). 

While Fricke dosimetry has been used as a tool to accurately assess radiation dose, its application to biochemical models has yet to be extensively studied. In a chemical model, iron is able to bring about both single-stranded and double-stranded DNA breaks induced by γ-IR in a concentration-dependent manner, suggesting that the iron content may be a critical catalyst in IR-induced DNA damage [81]. Because of the timescale on which these reactions occur, the oxidation events catalyzed by iron are likely site-specific. That is, redox-active iron, principally as Fe^2+^, is loosely bound to target molecules such as DNA. This Fe^2+^ than reacts with O_2_ or H_2_O_2_ to form oxidants right at that location, bringing about oxidative damage, for example to DNA [56]. 

Other iron metabolic perturbations induced by IR have been evaluated in other biologically relevant models. Like P-AscH^−^, IR has been shown in Chinese hamster fibroblast cells to bring about the oxidation of the [4Fe-4S]^2+^ cluster of aconitase [82]. In U373-MG cells, 5 Gy IR increased the expression of transferrin receptor 9–12 h after exposure [83]. This is consistent with the oxidation, and thus inactivation, of aconitase leading to the activation of iron response protein-1 (IRP1) [84,85]. This activation could further increase the iron content of cells and thereby increase the potential for more oxidative damage. 

In addition to modifying Fe-S clusters, IR can also interact with heme. In red blood cells, a significant, dose-dependent increase in methemoglobin has been observed following γ-IR, indicating oxidation of the heme center [86]. It has also been observed that IR is able to increase the amount of redox-active Fe in MIA PaCa-2 pancreatic tumor homogenates. This report also showed an IR-dose-dependent increase in redox-active Fe^2+/3+^; the addition of P-AscH^−^ further enhanced this effect. More recently, IR has been shown to induce ferroptosis or Fe-catalyzed lipid peroxidation leading to cell death in various lung adenocarcinoma, NSCLC, and glioma cell lines [87,88]. Therefore, P-AscH^−^-mediated iron metabolic perturbations are likely able to enhance radiosensitivity by increasing the redox-active pool of labile Fe^2+/3+^ following IR; this would further propagate damaging redox reactions, leading to enhanced site-specific damage to critical biomolecules, such as DNA.

## 6. P-AscH^−^ as a Radioprotector in Normal Tissue

As the data reviewed above indicate, P-AscH^−^ is a pro-oxidant in tumor cells via production of H_2_O_2_. However, it is neither cytotoxic towards normal tissue cells nor does it radiosensitize cells from normal tissues to IR (Figure 2). Moreover, there is mounting evidence that P-AscH^−^ may be radioprotective in cancer patients. Because P-AscH^−^ can act as an antioxidant in normal tissue, its reducing capacity may be a buffer to blunt the oxidative stress induced by chemoradiation in normal tissue [89,90]. The antioxidant effects of P-AscH^−^ have been purported to reduce chemoradiation side effects in clinical circumstances. In a study of breast cancer patients, treated weekly with chemoradiation or chemoradiation and P-AscH^−^ (intravenous dosage of 7.5 g weekly), the subjects in the group treated with P-AscH^−^ indicated significantly fewer side effects than those in the group treated with only chemoradiation. However, because of limitations in the study, whether normal tissue protection or improved treatment efficacy was the driving factor for these improved quality-of-life indicators was not determined [91].

It is interesting that, in the phase I clinical trial with pancreatic cancer patients (vide supra), five of the patients in the P-AscH^−^-plus-gemcitabine group experienced decreased systemic oxidative stress, evaluated by plasma F_2_-isoprostane levels, compared to baseline oxidative stress [30]. In a previous pancreatic cancer in vivo study, researchers found that P-AscH^−^ did not increase systemic changes and oxidative stress markers [4]. Importantly, P-AscH^−^ appeared to partially reverse radiation-induced injury in jejunal crypts [5]. These studies provide both direct and indirect data consistent with P-AscH^−^ being a radioprotector for normal tissue.

These data on the potential radioprotection of normal tissue by P-AscH^−^ are promising. More detailed studies are clearly warranted, addressing basic mechanisms and especially clinical potential [4,92,93].

## 7. Conclusions

From its first introduction in the 1970s, P-AscH^−^ has had a controversial history as an anti-cancer drug. However, in the past two decades, there has been significant progress in understanding its safety, efficacy, and mechanism in inducing selective radiosensitization of cancer cells while protecting normal cells. P-AscH^−^ has been shown in vitro and in vivo to be an efficient radiosensitizer in various cancer types including glioblastoma, NSCLC, sarcoma, and pancreatic cancer, while preventing or reversing normal tissue injury by acting as an antioxidant in tissues and systemically [4,5,55,61]. Additionally, the data presented in this review show synergistically increased DNA damage with combination treatment of RT and P-AscH^−^, associated with H_2_O_2_ formation. While there is ongoing research exploring the mechanism of P-AscH^−^-induced toxicity in cancer cells as well as its role in normal tissue physiology, the current body of literature spanning preclinical and clinical research support continued exploration into this novel radio-sensitization strategy for several aggressive and prognostically concerning cancer types. 

## 8. Materials and Methods

### 8.1. Catalase Activity Assay

Catalase activity was determined using UV–Vis spectroscopy by following the rate of removal (oxidation) of hydrogen peroxide due to catalase [94,95]. Hydrogen peroxide was monitored at 240 nm, ε_240_ = 39.4 M^−1^ cm^−1^ [96]. The assay was conducted in a 55.6 mM (pH 7.0) potassium phosphate buffer. The reaction was initiated by adding a 30 mM H_2_O_2_ solution (in working buffer) to bring the final H_2_O_2_ concentration to 10 mM in the cuvette. Immediately after adding H_2_O_2_, the kinetic analysis was started. The absorbance of hydrogen peroxide was monitored over 120 s at 10 s intervals. The rate of disappearance was then converted to a natural log to determine the *k*U of activity. The protein content (Lowry protein assay) in each assay was determined and used to normalize the activity of catalase per mg of protein (m*k* U/mg protein).

### 8.2. Lentiviral Transduction of U87 Cells with Catalase

The catalase-pTRIPZ vector was provided by the laboratory of Douglas Spitz [97]. To produce lentivirus, TSA201 cells were used along with VSV-G and psPAX2 helper vectors (Addgene, Watertown, MA, USA). The virus was collected from TSA201 cell cultures, centrifuged to remove cell debris, and filtered using 0.45 µm filters from the ZymoPURE^tm^ II Plasmid Midiprep Kit (Zymo Research, Irvine, CA, USA). U87 cells were plated and allowed to grow for 24 h, and then virus was added to the cells with 8 µg/mL of polybrene for a total of 48 h, with fresh virus being added after 24 h. Following transduction, the cells were selected with 2.5 µg/mL puromycin. The general population that survived puromycin selection was then validated for overexpression of catalase by treatment with 1 µg mL^−1^ doxycycline hyclate (Fisher Bioreagents BP2653-5, Geel, Belgium) for 48 h. 

### 8.3. γH2AX Staining and Flow Cytometry

U87 GBM cells were cultured in BR-15 cell culture medium (DMEM F12, FBS, sodium pyruvate, Penicillin/streptomycin, HEPES, insulin, FGF) in T-175 flasks at 37 °C and 21% O_2_. Once cells reached a confluency of 70%, the medium was removed, and the cells were harvested utilizing 0.25% trypsin–EDTA. Cells were counted using a Beckman Coulter Counter and then plated at 2.5 × 10^5^ cells per plate in 60 mm dishes in BR-15. After 24 h at 37 °C and 21% O_2_, the cells were treated with doxycycline (final concentration 1 μg/mL). After 48 h, the cells were divided in groups and subjected to different treatments. The P-AscH^−^ treatment was 2 mM (10 pmol/cell), and the RT dosage was 4 Gy. For the combination of P-AscH^−^ and RT, P-AscH^−^ was administered for 1 h prior to RT. After RT, the cells were incubated for 30 to 45 min, then were harvested utilizing 0.25% trypsin–EDTA, and finally were washed with cold PBS. Following this, they were resuspended in cold (−20 °C) 70% EtOH and incubated at −20 °C for 1 h before being placed at 4 °C for 48 h to fix them. After 48 h, the fixed cells were washed with PBS and rehydrated in cold TBST. Then, a primary antibody for anti-γH2AX purchased from Cell signaling (Cat#2577L) (rabbit polyclonal, 1:1000 diluted in TBST) was added to the fixed cells, which were incubated for 24 h at 4 °C. The primary antibody was removed, and the cells were washed with PBS (2% FBS) before applying the secondary antibody (FITC-conjugated goat anti-rabbit, purchased from Sigma Cat#F0382, diluted 1:300 in TBST) for 1 h in the dark. The cells were then washed with PBS and resuspended in 500 μL PBS for flow cytometry imaging. Mean Fluorescence Intensity (MFI) of 10,000 cells was collected, and autofluorescence was corrected for. Following data collection and analysis, paired *t*-tests were performed for statistical analysis.
